# Activation of PAD4 in NET formation

**DOI:** 10.3389/fimmu.2012.00360

**Published:** 2012-11-29

**Authors:** Amanda S. Rohrbach, Daniel J. Slade, Paul R. Thompson, Kerri A. Mowen

**Affiliations:** ^1^Department of Chemical Physiology, The Scripps Research InstituteLa Jolla, CA, USA; ^2^Department of Chemistry, The Scripps Research InstituteJupiter, FL, USA

**Keywords:** PAD4, citrullination, deimination, neutrophil, NET

## Abstract

Peptidylarginine deiminases, or PADs, convert arginine residues to the non-ribosomally encoded amino acid citrulline in a variety of protein substrates. PAD4 is expressed in granulocytes and is essential for the formation of neutrophil extracellular traps (NETs) via PAD4-mediated histone citrullination. Citrullination of histones is thought to promote NET formation by inducing chromatin decondensation and facilitating the expulsion of chromosomal DNA that is coated with antimicrobial molecules. Numerous stimuli have been reported to lead to PAD4 activation and NET formation. However, how this signaling process proceeds and how PAD4 becomes activated in cells is largely unknown. Herein, we describe the various stimuli and signaling pathways that have been implicated in PAD4 activation and NET formation, including the role of reactive oxygen species generation. To provide a foundation for the above discussion, we first describe PAD4 structure and function, and how these studies led to the development of PAD-specific inhibitors. A comprehensive survey of the receptors and signaling pathways that regulate PAD4 activation will be important for our understanding of innate immunity, and the identification of signaling intermediates in PAD4 activation may also lead to the generation of pharmaceuticals to target NET-related pathogenesis.

## THE PEPTIDYL ARGININE DEIMINASE FAMILY

**FIGURE 1:**
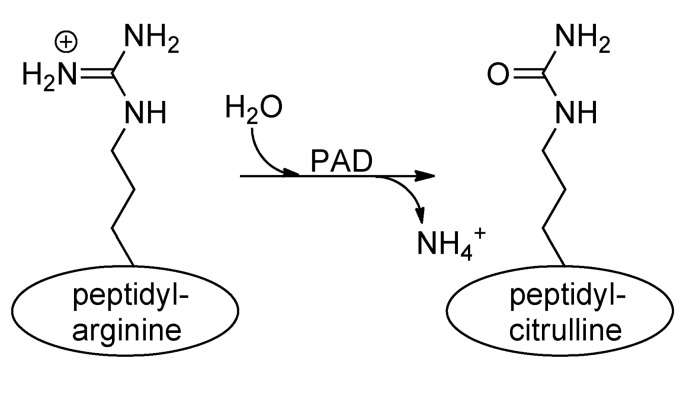
**Conversion of arginine into citrulline**.Peptidylarginine deiminase (PAD) enzymes convert an arginine residue into citrulline.

The mammalian genome encodes 20 natural amino acids; however, this diversity is greatly increased by posttranslational modification of the original set to yield more than one hundred unique amino acids (Uy and Wold, 1977). Citrullination, or deimination, is the posttranslational modification of an arginine to a citrulline residue. Hydrolysis of the guanidino group of the arginine yields a ureido group and the loss of an ammonia (**Figure 1**). Citrullination is catalyzed by the peptidyl arginine deiminase family of enzymes, or PADs. This process results in the loss of positive charge and an approximately 1 Da increase in mass. While this modification seems quite modest, the loss of positive charge, and hydrogen bond acceptors, can have dramatic effects on cell signaling because these types of interactions are critical for stabilizing protein–protein, protein–DNA, and protein–RNA interactions. Additionally, this PTM may disrupt intra-molecular interactions, which could trigger major conformational changes in a protein, potentially altering intermolecular interactions and decreasing protein stability (Vossenaar et al., 2003).

Five PAD enzymes are expressed in humans and mice, and the major difference between these isozymes appears to be tissue localization. PADs 1, 3, and 6 are expressed in the skin and uterus, hair follicles, and egg, ovary and embryo, respectively (Vossenaar et al., 2003; Wang et al., 2012). PAD4 expression has been reported in granulocytes, as well as some cancerous cell lines and tumors (Vossenaar et al., 2003; Chang et al., 2009; Jones et al., 2009; Hemmers et al., 2011; Wang et al., 2012), and most recently in mammalian oocytes and the preimplantation embryo (Brahmajosyula and Miyake, 2011). PAD2 has a much broader tissue expression profile and can be found in the CNS, skeletal muscle, and cells of the immune system (Vossenaar et al., 2003). PADs 1, 2, and 4 are the only PADs expressed in the hematopoietic lineage, and, thus, are especially of immunological interest.

*PADI* genes, encoding the PAD enzymes, are located in a single gene cluster on chromosome 1p36.1 in humans and chromosome 4pE1 in mice (Vossenaar et al., 2003). The regions encoding the *PADI* locus in humans and mice span 334.7 and 230.4 kb, respectively (Vossenaar et al., 2003). All PAD enzymes are highly conserved, sharing at least 50% sequence homology among isozymes and 70% or greater homology of each vertebrate ortholog (Vossenaar et al., 2003). Eighteen per cent sequence identity is shared among all PADs (Vossenaar et al., 2003). Catalysis by all of these enzymes is calcium dependent, and, at least *in vitro*, requires calcium concentrations that are higher than that available in homeostatic cytoplasm, indicating calcium flux or a calcium-producing event is necessary to induce activity (Arita et al., 2004; Kearney et al., 2005; Raijmakers et al., 2007; Knuckley et al., 2010). Alternatively, a PTM or interacting protein may decrease the calcium concentration required for activation to physiologic levels.

## PAD4

Of all of the PADs, PAD4 is of specific interest because of its importance in innate immunity and its putative role in a variety of pathogenic states, including autoimmune diseases, such as rheumatoid arthritis (RA), multiple sclerosis (MS), ulcerative colitis (UC), and systemic lupus erythematosus (SLE), and other inflammatory conditions, such as sepsis and thrombosis (Jones et al., 2009). Many autoantibodies in RA are directed against citrullinated proteins. In fact, the presence of anti-citrullinated protein antibodies (ACPA) is a better predictor of RA than rheumatoid factor (Vossenaar et al., 2003), and, in 2011, ACPA were included in the new classification criteria for RA (Willemze et al., 2012). A genome-wide association study identified a PAD4 haplotype that is associated with RA in a Japanese population, albeit with a low odds ratio (OR = 1.14; Suzuki et al., 2003). The mutations in the PAD4 gene appear to confer prolonged stability to the transcript, leading to a model where increased expression of PAD4 in these populations would favor the generation of citrullinated self-epitopes to prime the autoimmune response (Suzuki et al., 2003). Though this association has been confirmed in other Asian populations, it has not been replicated in studies using patients from all Western European populations, indicating that the RA-associated PAD4 haplotype found in Asian RA patients can not explain the presence of ACPA’s in all ethnicities (Klareskog et al., 2008). Interestingly, the PAD4 RA-associated disease haplotype has also been found in some Japanese patients with UC (Chen et al., 2008). MS patients have increased levels of the citrullinated form of myelin basic protein (Wood et al., 1996; Moscarello et al., 2007), and both PAD2 and PAD4 are overexpressed in the brains of MS patients (Wood et al., 2008). Finally, as will be discussed later in this review, in response to microbes, neutrophils can extrude their nuclear contents to form antimicrobial neutrophil extracellular traps (NETs; Brinkmann and Zychlinsky, 2007). Since PAD4 is essential for the formation of NETs (Li et al., 2010; Hemmers et al., 2011), PAD4 has also been implicated in NET-related pathologies, such as SLE and thrombosis, where NETs presumably promote deleterious inflammatory responses (Kessenbrock et al., 2009; Logters et al., 2009; Fuchs et al., 2010; Hakkim et al., 2010; Garcia-Romo et al., 2011; Villanueva et al., 2011). Thus, PAD4 may be a relevant target for several disease indications.

PAD4 is a 74 kDa protein that exists as a head-to-tail dimer (Arita et al., 2004; Liu et al., 2011). Each monomer consists of two N-terminal immunoglobulin (Ig) domains, formed by Ig subdomain 1, which contains nine β-sheets, and Ig subdomain 2, which contains 10 β-sheets and four short α-helices. The C-terminal catalytic domain adopts the α/β propeller fold that is characteristic of the deiminase superfamily (Shirai et al., 2001; Arita et al., 2004). The C-terminal catalytic domain is the most highly conserved area of the molecule (Vossenaar et al., 2003), suggesting that the active sites are likely quite similar among PADs. While a high degree of conservation exists among PADs, PAD4 is the only family member to contain a classic nuclear localization sequence (56-PPAKKKST-63), found in Ig1 near the N-terminus, and, thus, is trafficked to the nucleus (Nakashima et al., 2002; Vossenaar et al., 2003; Arita et al., 2004). However, it is worth noting that recent data indicates that other PADs, most notably PAD2, are localized to the nucleus (Zhang et al., 2012).

PAD4 binds five calcium molecules, designated Ca1–Ca5, in a cooperative manner (Arita et al., 2004; Kearney et al., 2005; Liu et al., 2011). Ca1 and Ca2 bind in the C-terminal catalytic domain, and their binding induces major conformational changes that move several active site residues into positions that are competent for catalysis (Arita et al., 2004). This calcium-induced formation of the active site is unique to the PADs, and represents a novel mechanism of enzyme activation (Arita et al., 2004). Calcium binding also induces large structural changes in the N-terminus of the protein. For example, binding of Ca3, Ca4, and Ca5, along with two water molecules, induces the formation of the a1 α-helix, which is disordered in the apoenzyme (Arita et al., 2004). These conformational changes may provide, or remove, docking sites for other proteins, which may serve to further regulate PAD activity.

## BIOCHEMICAL ACTIVATION OF PAD4

While it is unknown whether all PAD enzymes are capable of multimerizing, the dimer has been suggested to be required for PAD4 activity (Arita et al., 2004; Liu et al., 2011). However, the effects on enzyme activity and the calcium dependence of the enzyme are relatively minor (approximately twofold), and we routinely see robust enzyme activity at concentrations of protein that are an order of magnitude below the *K*_d_ of the dimer (Kearney et al., 2005). Nevertheless, dimer formation may represent a possible regulatory mechanism (Liu et al., 2011). Dimerization is mediated by both hydrophobic interactions and salt bridges between adjacent monomers (Arita et al., 2004; Liu et al., 2011).

The PADs display limited substrate specificity and citrullinate many proteins *in vitro*, preferring to modify arginine residues present in unstructured regions; the rate of substrate turnover is inversely proportional to the structural order of the substrate (Arita et al., 2006; Knuckley et al., 2010). Structurally, PAD4 interacts with the backbone atoms surrounding the site deimination, i.e., R-2, R-1, R0, and (R + 1), with few, if any, contacts with the side chains (Arita et al., 2006). The only requirement appears to be a small side chain at the R-2 position so as to avoid steric contacts with the active site (Arita et al., 2006). Upon binding to PAD4 the backbone of the substrate adopts a β-turn-like conformation within the substrate binding cleft (Arita et al., 2006), thereby explaining why the enzymes show such a high level of substrate promiscuity. In contrast to the situation *in vitro*, the PADs are believed to show greater substrate specificity *in vivo.* Presumably, interacting proteins modulate the substrate specificity of the enzyme or spatially target the enzyme to specific regions of the cell. For example, PAD4 is present in the nucleus and may be targeted to chromatin where it citrullinates a number of nuclear proteins, including the histones and protein arginine methyltransferase 1 (PRMT1; Vossenaar et al., 2003; Slack et al., 2011b). Although PAD4 was reported to convert monomethylated arginine residues to citrulline (Wang et al., 2004), this modification occurs at rates that are 10^2^- to 10^3^-fold slower than an unmodified arginine, suggesting that the, so-called “demethylimination” reaction is not physiologically relevant (Hidaka et al., 2005; Kearney et al., 2005; Raijmakers et al., 2007; Thompson and Fast, 2006) and that citrullination simply antagonizes arginine methylation as originally suggested by Cuthbert et al. (2004).

In addition to the aforementioned protein substrates, PAD4, as well as the other PADs, autocitrullinate at several sites on the enzyme (Andrade et al., 2010; Mechin et al., 2010; Slack et al., 2011a). Although autocitrullination has been reported to directly modulate PAD4 activity (Andrade et al., 2010; Mechin et al., 2010), in our hands, this self-modification has no direct effect on enzyme activity, but it does appear to modulate protein–protein interactions (Slack et al., 2011b). For example, Slack et al. (2011b) demonstrated that citrullination of PAD4 reduces its ability to interact with PRMT1 and histone deacetylase (HDAC) 1, perhaps modulating its ability to alter gene transcription.

## PAD MECHANISM AND INHIBITION

Given the substrate promiscuity of the PADs, it is unsurprising that the PADs also citrullinate a number of small molecule arginine mimics, including benzoyl arginine ethyl ester (BAEE) and benzoyl arginine amide (BAA). In fact, these compounds have served as important mechanistic probes of PAD4 catalysis and provided the molecular scaffold for the construction of the first highly potent PAD4 inhibitors. Below we highlight key mechanistic insights that guided the design of these inhibitors.

**FIGURE 2:**
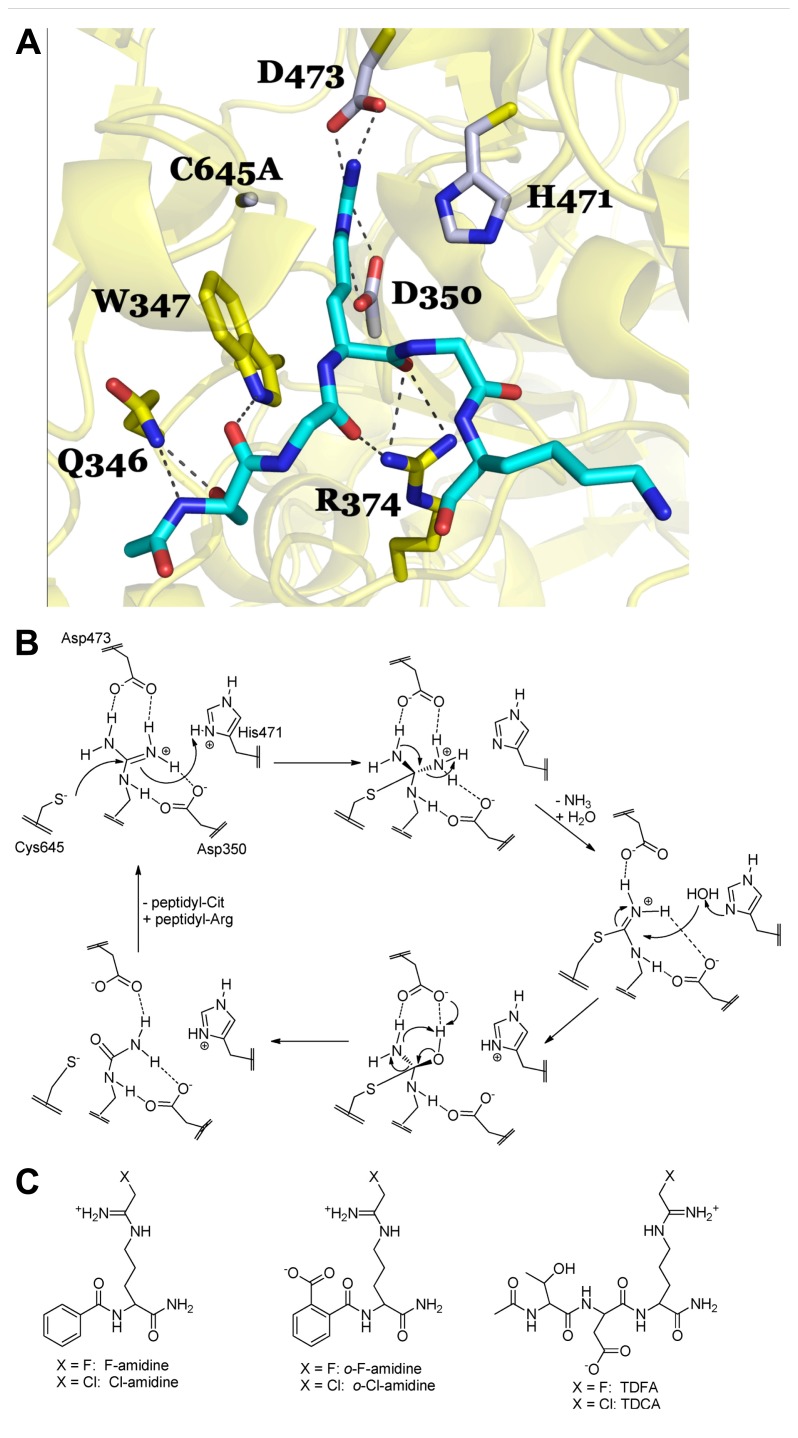
**(A)** PAD4 in complex with Histone H4 1–5 (SGRGK). PAD4 active site residue side chains are colored gray (D350, H471, D473, C645A) and residues that are involved in binding the H4 1–5 backbone and S1 are colored yellow (Q346, W347, R347). N-terminally acetylated H4 1–5 is shown in cyan with Arginine 3 bound in the PAD4 active site. Polar residue interactions between PAD4 and H4 1–5 are indicated by dashed lines. The mutation of the active site cysteine residue (C645) to an alanine (C645A) was necessary to achieve substrate binding in the crystal structure as described in Arita et al. (2006). Figure was created from the structure filed under PDB code 2dey. **(B)** Proposed catalytic mechanism for PAD4. **(C)** Haloacetamidine-based inhibitors targeting the PAD isozymes.

Briefly, there are four key catalytic residues, Asp350, His471, Asp473, and Cys645. Asp473 binds to both ω-nitrogens and Asp350 coordinates to one ω-nitrogen and the δ-nitrogen (**Figure 2A**). Cys645 and His471 lie on opposite sides of the guanidinium group and are appropriately positioned to promote catalysis via nucleophilic attack on the guanidinium carbon (Cys645) and protonation of the developing tetrahedral intermediate (His471; **Figure 2B**). Collapse of this intermediate leads to the loss of ammonia and the formation of the stable *S*-alkyl thiouronium intermediate that is subsequently hydrolyzed via a second tetrahedral intermediate to form citrulline; His471 likely activates the water molecule for nucleophilic attack (**Figure 2B**). Mechanistic studies (Knuckley et al., 2007, 2010), including mutagenesis, pH rate profile, solvent isotope effects, and solvent viscosity effects, as well as crystal structures of PAD4 bound to several substrates (i.e., BAA; Arita et al., 2006), and histone H3 and histone H4 tail analogs; Arita et al., 2004, 2006), and inhibitors (i.e., F-amidine, Cl-amidine, *o*-F-amidine, *o*-Cl-amidine, and TDFA; Luo et al., 2006a; Causey et al., 2011; Jones et al., 2012), provide strong support for the above mechanism and helped drive our thoughts about inhibitor design. For example, the presence of a reactive active site Cys prompted us to consider the synthesis of irreversible inhibitors (Knuckley et al., 2007, 2010). Additionally, the fact that mono-methylated arginine residues were exceptionally poor PAD substrates, as well as the steric restraints of the active site (Arita et al., 2004; Kearney et al., 2005; Raijmakers et al., 2007), told us that the reactive moiety would have to be relatively isosteric with respect to the substrate guanidinium. Furthermore, mutagenesis studies on the two active site aspartyl groups in PAD4, Asp350 and Asp473, showed that these residues are critical for catalysis (Knuckley et al., 2007), indicating that proper positioning, hydrogen bonding, and electrostatic interactions between these residues and the substrate guanidinium are critical determinants for efficient substrate turnover, and would have to be maintained for efficient enzyme inactivation. As such, we initially focused our efforts on synthesizing F-amidine and Cl-amidine (**Figure 2C**), two haloacetamidine-containing compounds that we hypothesized, and later confirmed, would inactivate the PADs by alkylating Cys645 (Luo et al., 2006a,b). These initial inhibitors, F-amidine and Cl-amidine, as well as our second generation compounds *o*-F-amidine, *o*-Cl-amidine, and TDFA (Knuckley et al., 2010; Causey et al., 2011), which show enhanced potency and selectivity, are bioavailable and have been used to show that the PADs play important roles in controlling gene transcription (Li et al., 2008; Yao et al., 2008; Jones et al., 2012; Zhang et al., 2011, 2012), fertility (Kan et al., 2012), differentiation (Slack et al., 2011c), and NET formation (Wang et al., 2009; Li et al., 2010). Additionally, Cl-amidine, or a Cl-amidine analog, decrease disease severity in animal models of RA, UC, nerve damage, and cancer (Chumanevich et al., 2011; Lange et al., 2011; Willis et al., 2011; Wang et al., 2012). Specifically, we were the first to show that the PAD inhibitor, Cl-amidine dose dependently decreased inflammation by up to 55% in the mouse collagen-induced arthritis (CIA) model of RA. Concomitant with the decreased severity there were significant decreases in the levels of citrullinated proteins, complement deposition, and epitope spreading (Willis et al., 2011). Similar dose-dependent effects were observed in the dextran sodium sulfate (DSS) model of UC where dosing of up to 75 mg/kg after the onset of disease led to significant reductions in weight loss, inflammation score, and colon lengthening (Chumanevich et al., 2011). The effects of Cl-amidine on nerve damage was examined in a chick embryo model of spinal cord injury where treatment with Cl-amidine reduced the abundance of deiminated histone 3, consistent with inhibition of PAD activity, and significantly reduced apoptosis and tissue loss following injury at embryonic day 15 (Lange et al., 2011). Finally, Wang et al. (2012) showed that a Cl-amidine analog, YW3-56, decreased tumor growth in a mouse sarcoma S-180 cell-derived solid tumor model and that additive effects on growth inhibition were observed when this compound was combined with the histone deacetylase inhibitor SAHA. We have observed similar effects with Cl-amidine in xenografts model of ductal carcinoma *in situ* (McElwee et al., 2012).

## THE FUNCTION OF PAD4 IN NEUTROPHILS

Neutrophils are terminally differentiated granulocytes, which differentiate from hematopoietic stem cells in the bone marrow, and make up to 75% of white blood cells in the circulation (Ermert et al., 2009b). Mature neutrophils are released into circulation, where they have a very short life span of several hours to one day before undergoing apoptosis (Borregaard, 2010). Neutrophils are an important component of the innate immune system and form the first line of defense against invading pathogens, such as bacteria and fungi (Borregaard, 2010). Neutrophils contain an arsenal of antimicrobial proteins and peptides in primary (or azurophilic), secondary (or specific), and tertiary granules. Primary granules are mostly composed of proteases, such as myeloperoxidase and neutrophil elastase (NE), and antimicrobial peptides, such as defensins (Ermert et al., 2009b). Secondary and tertiary granules contain lactoferrin and gelatinase, respectively (Mantovani et al., 2011). Secretory granules also harbor stores of membrane proteins, such as the NADPH oxidase machinery (Nox; Borregaard, 2010), which can be trafficked to the surface of the cell quickly when necessary. In response to chemoattractants, neutrophils are guided to areas of infection, where they respond with several effector mechanisms to invading pathogens, including phagocytosis, release of bactericidal products, and ROS production (Borregaard, 2010). Neutropenia, or the state of having too few neutrophils, leads to extreme immunosuppression and susceptibility to bacterial infections, which can be fatal (Janeway, 2005).

In 2004, Brinkmann et al. (2004) recognized the formation of NET structures, which were extruded by neutrophils in response to bacteria. NETs are composed of nuclear DNA that are decorated with a variety of nuclear and granular proteins, actively thrown out into the extracellular space, and result in death of the NET-producing cell (Brinkmann et al., 2004). Cell death by this mechanism is unique from apoptosis and necrosis and has been termed “NETosis”(Gupta et al., 2010). NETs ensnare extracellular bacteria, which are killed by the inherent antimicrobial properties of NET-associated proteins, such as histones (Parseghian and Luhrs, 2006), NE (Papayannopoulos and Zychlinsky, 2009), and lactoferrin (Papayannopoulos and Zychlinsky, 2009). These structures represent a novel method for pathogen killing, independent of both degranulation and phagocytosis, and have been shown to effectively kill a variety of pathogens, including bacteria, fungi, and protozoa (Papayannopoulos and Zychlinsky, 2009; Remijsen et al., 2011a). NETs have also been reported to occur in response to viral infection; however, they not appear to show any observable anti-viral capabilities (Hemmers et al., 2011). NETs may represent a killing mechanism for pathogens too large for the neutrophil to phagocytose, such as fungal hyphae or helminthes (Urban et al., 2006). Interestingly, bacteria have adapted defense mechanisms to NET formation. For example, the Group A *Streptococcus *express a DNase enzyme that can degrade NETs (Buchanan et al., 2006), and *Pseudomonas aeruginosa *expresses surface molecules that can prevent neutrophil activation and NET formation (Khatua et al., 2012).

Histone citrullination is thought to promote NET formation by inducing chromatin decondensation and facilitating the expulsion of chromosomal DNA coated with antimicrobial molecules (Neeli et al., 2008; Wang et al., 2009; Li et al., 2010). In fact, chemical inhibition of PAD4 activity significantly reduces histone decondensation and NET formation in response to either the calcium ionophore ionomycin or the bacterium *Shigella flexneri *(Wang et al., 2009). Our group and the Wang group have independently created PAD4-deficient mice (Li et al., 2010; Hemmers et al., 2011). Neutrophils isolated from PAD4-deficient mice are unable to citrullinate histones, decondense chromatin, and generate NETs (Li et al., 2010; Hemmers et al., 2011). Because of their inability to form NETs, PAD4 KO mice were shown to be more susceptible to bacterial infection (Li et al., 2010), and, thus, PAD4 is an important mediator of innate immunity.

Neutrophil elastase, a neutrophil serine protease, resides in the azurophilic granules and is a component of NETs (Borregaard, 2010; Amulic et al., 2012). The cleavage of microbial virulence factors by NE is essential for the clearance of specific Gram-negative bacteria (Pham, 2006). NE also cleaves histones to drive chromatin decondensation during NET formation (Papayannopoulos et al., 2010). Indeed, NE is essential for the process of NETosis (Papayannopoulos et al., 2010), and it is interesting to speculate that histone citrullination, by PAD4, promotes a relaxing of the chromatin structure, allowing NE to gain access. Thus, the activity of NE and PAD4 may converge upon the chromatin decondensation process and NET formation. Neutrophils isolated from PAD4-deficient mice will be useful to delineate the hierarchy between PAD4 and other molecules, like NE, that are required for NETosis.

## STIMULATION OF PAD4 ACTIVITY

A number of stimuli, including live and heat-killed bacteria, fungi, protozoa, and chemokines have been reported to induce NET formation (Guimaraes-Costa et al., 2012). Because NET formation is PAD4-dependent (Li et al., 2010; Hemmers et al., 2011), these same stimuli likely also induce PAD4 activation. However, the activity of PAD4 in relation to each stimuli must be assessed by looking for citrullination of histones, which is both a hallmark of PAD activity (Neeli et al., 2008, 2009) and a component of NETs (Li et al., 2010; Hemmers et al., 2011). Only a handful these, including live bacteria, the Gram-negative bacterial cell wall component lipopolysaccharide (LPS), the Gram-positive bacterial cell wall component lipoteichoic acid (LTA), the fungal cell wall component zymosan, the proinflammatory cytokine TNFα, and H_2_O_2_ have been shown to induce PAD4 activity and histone citrullination (Neeli et al., 2008, 2009; Li et al., 2010; Hemmers et al., 2011). As discussed earlier, PAD4 is calcium-dependent, and it is thought that PAD4 requires calcium levels higher than are found in the homeostatic cell to be active (Vossenaar et al., 2004). Not surprisingly, the calcium ionophore ionomycin activates PAD4 to induce histone citrullination and elicit NET formation (Neeli et al., 2008, 2009; Wang et al., 2009; Li et al., 2010). **Table 1** catalogs the variety of stimuli reported to stimulate NET formation.

**Table 1:** NET/PAD4 stimuli.

NET stimuli	Shown to activate PAD4	Reference
Activated endothelial cells	n.d.	Gupta et al. (2010)
*Aspergillus fumigatus*	n.d.	Bruns et al. (2010)McCormick et al. (2010)
*Candida albicans*	n.d.	Ermert et al. (2009a), Urban et al. (2009), Yost et al. (2009), Hakkim et al. (2010), Svobodova et al. (2012)
Opsonized *Candida albicans*	n.d.	Metzler et al. (2011)
*Cryptococcus *species**	n.d.	Urban et al. (2009), Springer et al. (2010)
*Escherichia coli*	Yes	Grinberg et al. (2008)), Wang et al. (2009), Yost et al. (2009), Yan et al. (2012)
f-MLP	Yes	Neeli et al. (2008)
H_2_O_2_	Yes	Neeli et al. (2008), Li et al. (2010)
*Haemophilus influenzae*	n.d.	Juneau et al. (2011)
IL-8+ *Shigella flexneri*	Yes	Wang et al. (2009)
IL-8^1^	n.d.	Brinkmann et al. (2004), Gupta et al. (2005)
Ca^2^^+^ ionophore	Yes	Wang et al. (2009)
*Klebsiella pneumoniae*	n.d.	Papayannopoulos et al. (2010)
*Leishmania *species**	n.d.	Guimaraes-Costa et al. (2009), Gabriel et al. (2010)
*Listeria monocytogenes*	n.d.	Ermert et al. (2009a), Munafo et al. (2009)
LPS	Yes	Neeli et al. (2008), Li et al. (2010), Munafo et al. (2009)
Lipoteichoic acid	Yes	Neeli et al. (2009)
*Mycobacterium* species**	n.d.	Ramos-Kichik et al. (2009)
Nitric Oxide	n.d.	Patel et al. (2010)
Platelet activating factor	n.d.	Yost et al. (2009), Fuchs et al. (2010)
Platelet TLR-4	n.d.	Clark et al. (2007)
Phorbol-12-myristrate-13-acetate (PMA)	Yes	Brinkmann et al. (2004), Ermert et al. (2009a), Munafo et al. (2009), Neeli et al. (2009), Chow et al. (2010), Li et al. (2010), Papayannopoulos et al. (2010), Pilsczek et al. (2010), Lin et al. (2011), Metzler et al. (2011), Remijsen et al. (2011b), Villanueva et al. (2011), Menegazzi et al. (2012), Palmer et al. (2012), Parker et al. (2012), Yan et al. (2012)
*Pseudomonas aeruginosa*	n.d.	Pilsczek et al. (2010)
*Salmonella typhimurium*	n.d.	Brinkmann et al. (2004)
*Shigella flexneri*	Yes	Brinkmann et al. (2004), Li et al. (2010)
*Staphylococcus aureus*	n.d.	Brinkmann et al. (2004), Chow et al. (2010), Pilsczek et al. (2010)
Opsonized *Staphylococcus aureus*	n.d.	Palmer et al. (2012)
*Staphylococcus epidermidis* δ-toxin**	n.d.	Cogen et al. (2010)
*Streptococcus *species**	Yes^2^	Beiter et al. (2006), Buchanan et al. (2006), Lauth et al. (2009), Oehmcke et al. (2009), Crotty Alexander et al. (2010), Li et al. (2010), Pilsczek et al. (2010)
*Streptococcus pneumoniae* α-Enolase**	n.d.	Mori et al. (2012)
TNFα	Yes	Neeli et al. (2009), Wang et al. (2009)
*Toxoplasma gondii*	n.d.	Abi Abdallah et al. (2012)
*Yersinia enterocolitica*	n.d.	Casutt-Meyer et al. (2010)
Zymosan	Yes	Neeli et al. (2009)

*n.d., not determined.

1 Some investigators have reported that IL-8-induced NET formation may be sensitive to cell culture conditions (Marcos et al., 2011).

2 M1 GAS deficient in an extracellular DNase (Sda1) could induce PAD4-dependent NETs (Li et al., 2010).

Although little is known about the downstream signaling pathways required for PAD4 activation in neutrophils, cytoskeletal activity may be involved in PAD4 activation. Pretreatment of cells with nocodazole or cytochalasin D, which inhibit microtubule polymerization, prior to LPS stimulation leads to a reduction of histone citrullination and NET formation (Neeli et al., 2009). Additionally, blockade of integrin signaling through Mac-1 and cytohesin-1 impeded PAD4 activity and NET formation (Neeli et al., 2009). How cytoskeletal signaling impacts PAD4 is unknown; however, it has been proposed that the same receptors establish whether a cell will undergo phagocytosis or NET formation (Neeli et al., 2009). Indeed, studies have indicated that neutrophils initiate NET formation when phagocytosis of a large particle fails (Urban et al., 2006). Perhaps cytoskeletal activity and PAD4-mediated citrullination are linked because the initiation of NET formation represents a back-up killing mechanism following unsuccessful phagocytosis.

## PAD4 ACTIVITY AND ROS

The generation of reactive oxygen species (ROS) is initiated by a wide variety of neutrophil stimuli, including phagocytosis of pathogens and signaling by LPS and TNF (Kohchi et al., 2009), which are also NET-inducing stimuli. Indeed, ROS generation is required for NET formation, and, thus, it is likely that ROS generation is required for PAD4 activation as well. In neutrophils, superoxide (${\text{O}}_{2}^{•-}$) is generated by the NADPH complex (Nox) and by the electron transport chain in mitochondria (Kohchi et al., 2009). ${\text{O}}_{2}^{•-}$ is then converted to H_2_O_2_ spontaneously or by the enzyme superoxide dismutase (SOD; Kohchi et al., 2009). H_2_O_2_ acts directly on target cells and is converted to additional effectors by enzymes such as myeloperoxidase (MPO). Interestingly, the addition of H_2_O_2_ to primary murine or human neutrophils induces PAD4-dependent histone citrullination (Neeli et al., 2008; Li et al., 2010). ROS molecules are highly cytotoxic and act as antimicrobial agents, but they can also play a dual role as reversible signal transduction mediators to regulate redox-sensitive target proteins (Amulic et al., 2012).

**FIGURE 3:**
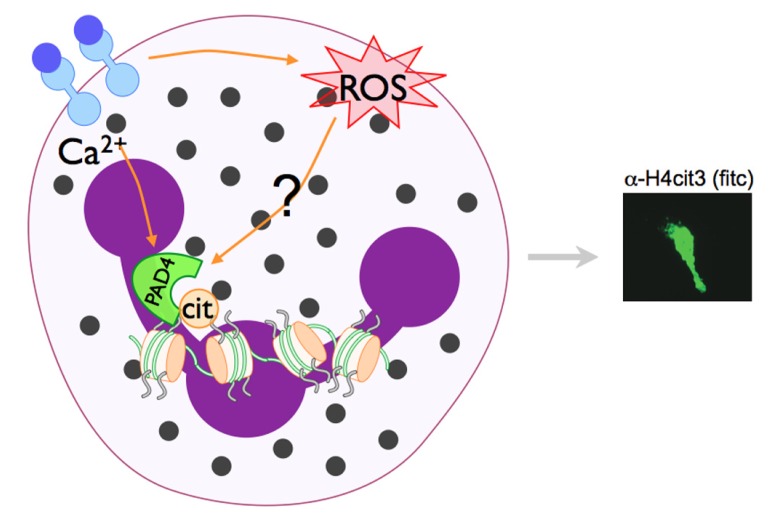
** Model of PAD4 activation in NET formation**. Pathways that activate NET formation are less defined than phagocytic pathways, but are known to require NADPH oxidase activity and the activation of PAD4 and subsequent histone citrullination. PAD enzymes are Ca^2+^-dependent. Since PAD4-mediated histone citrullination is abrogated by the NADPH inhibitor apocyanin (Neeli et al., 2009), we speculate that NADPH regulated ROS generation and increase Ca^2+^ levels may converge to activate PAD4 in neutrophils.

The link between ROS and NET formation was first recognized by the fact that patients with chronic granulomas disease (CGD), who are missing the Nox2 protein essential for NADPH assembly and, thus, cannot form ROS. Neutrophils isolated from CGD patients do not make NETs in response to *S. aureus* or phorbol myristate acetate (PMA; Fuchs et al., 2007). This phenotype is rescued by addition of H_2_O_2_ or exogenous glucose oxidase, which generates H_2_O_2_ (Fuchs et al., 2007), indicating that the ROS production facilitated by Nox2 is necessary for NETs. Catalase removes intracellular H_2_O_2_ by reduction to water and oxygen (Kohchi et al., 2009), and catalase inhibition increases intracellular H_2_O_2_ leading to increased NET production in healthy neutrophils (Fuchs et al., 2007). Subsequent studies have demonstrated that ROS generation is upstream of chromatin decondensation (Remijsen et al., 2011b), suggesting that NADPH oxidase activation may also be a prerequisite for PAD4 activation. Indeed, LPS-induced citrullination of histone H4 is decreased when cells are pre-incubated with the NADPH oxidase inhibitor apocynin (Neeli et al., 2009). Although, to our knowledge, the activity of PAD4 in CGD neutrophils has not yet been directly explored, since chromatin decondensation is not observed in CGD neutrophils, we would predict PAD4-mediated histone citrullination is also impaired. Since H_2_O_2_ treatment can activate PAD4-mediated histone deimination in primary murine and human neutrophils (Neeli et al., 2008; Li et al., 2010), and since NADPH activation seems to be upstream of NET formation (Neeli et al., 2009), we speculate that PAD4 activation may be downstream of NADPH (**Figure 3**).

The contribution of specific ROS molecules to NET formation has also been examined. NADPH oxidase or mitochondrial ROS selective inhibitors established a requirement for NADPH oxidase generated ${\text{O}}_{2}^{•-}$ (Nishinaka et al., 2011), but excluded a need for mitochondrial ROS in PMA-induced NET generation (Kirchner et al., 2012). MPO catalyzes the oxidation of Cl^-^ ions to generate hypochlorous acid (HOCl) and other reactive products using H_2_O_2_ as a cosubstrate (Arnhold and Flemmig, 2010). In the absence of MPO activity, NET generation is absent (Papayannopoulos et al., 2010; Metzler et al., 2011; Kirchner et al., 2012), but this phenotype can be rescued by addition of HOCl (Palmer et al., 2012). In fact, the HOCl product of MPO has also been found to be both necessary and sufficient for NET formation, and in CGD neutrophils, the addition of HOCl is also sufficient to initiate NET formation (Palmer et al., 2012). Taurine is a cellular antioxidant capable of reducing HOCl and H_2_O_2_ to promote cell survival (Palmer et al., 2012). Accordingly, preincubation of neutrophils with taurine prior to PMA or HOCl stimulation reduces NET formation (Palmer et al., 2012). Additionally, while SOD inhibition does not impede NET formation, addition of exogenous SOD does seem to increase NET production, perhaps owing to the increase in available H_2_O_2_ (Palmer et al., 2012). These studies indicate a model in which NADPH oxidase activity generates ${\text{O}}_{2}^{•-}$, which then dismutates to H_2_O_2_ either spontaneously or with the help of SOD, and is then used by MPO to generate HOCl, which is necessary and sufficient to induce NET formation. It will be interesting to determine whether HOCl can also directly regulate PAD4 activation.

## PAD4 AND AUTOPHAGY

Like ROS, autophagy has been shown to be required for chromatin decondensation during NET generation (Remijsen et al., 2011b); however, these two processes seem to be independent of each other (Remijsen et al., 2011b). Blockade of PI3K with wortmannin inhibits autophagy, and pretreatment of PMA stimulated neutrophils with wortmannin prevented chromatin decondensation (Remijsen et al., 2011b). However, no direct role between autophagy, PI3K and citrullination has been shown. Recently, newly developed PAD4 inhibitors were found to reduce autophagy processes in an osteosarcoma cell line (Wang et al., 2012), further providing evidence of a link between PAD4 activity and autophagy.

## CONCLUSION

Neutrophil extracellular traps have been reported in several pathological scenarios, including SLE, sepsis, thrombosis, and infectious disease, and may induce or exacerbate inflammation through prolonged inflammatory response, tissue damage, and presentation of neo-antigens. Indeed, our group recently described the PAD4-dependent formation of NETs in a murine model of the effector phase of RA; however, PAD4 was dispensable for disease in this model (Rohrbach et al., 2012). Pathology caused or exacerbated by NETs is an expanding field of research. Because PAD4 is required for NET formation, inhibition of PAD4 activity may improve clinical outcomes in patients experiencing these inflammatory diseases. In fact, PAD inhibitors have demonstrated efficacy in a variety of immune pathologies (Chumanevich et al., 2011; Lange et al., 2011; Slack et al., 2011c; Wang et al., 2012; Willis et al., 2011). Of course, because PAD4 is required for microbial-induced NET formation (Li et al., 2010; Hemmers et al., 2011), the side effects of PAD4-targeted therapeutics may also include increased susceptibility to infectious diseases.

PAD4 is an important component in the innate immune system, however its activity has been linked to a wide variety of disease states, including cancer, autoimmunity, and other inflammatory conditions. Despite its significance, little is understood about how the PAD4 enzyme becomes active in order to impart its helpful and harmful effects. At the protein level, calcium binding, dimerization, and autocitrullination may help regulate its activity. ROS may also play a role in regulating PAD4 activation (see model in **Figure 3**), and recently associations between PAD4 activity and autophagy have been proposed. Despite these efforts, much is left to understand about PAD4 enzyme regulation. Anti-PAD4 therapies have been proposed for inflammatory conditions and cancer, thus a more comprehensive understanding of the pathways that activate PAD4 in neutrophils will be important.

## Conflict of Interest Statement

The authors declare that the research was conducted in the absence of any commercial or financial relationships that could be construed as a potential conflict of interest.
